# Review of Recent Advances in Gas-Assisted Focused Ion Beam Time-of-Flight Secondary Ion Mass Spectrometry (FIB-TOF-SIMS)

**DOI:** 10.3390/ma16052090

**Published:** 2023-03-03

**Authors:** Agnieszka Priebe, Johann Michler

**Affiliations:** Empa, Swiss Federal Laboratories for Materials Science and Technology, Laboratory for Mechanics of Materials and Nanostructures, Feuerwerkerstrasse 39, CH-3602 Thun, Switzerland

**Keywords:** FIB-TOF-SIMS, gas injection system, elemental characterization

## Abstract

Time-of-flight secondary ion mass spectrometry (TOF-SIMS) is a powerful chemical characterization technique allowing for the distribution of all material components (including light and heavy elements and molecules) to be analyzed in 3D with nanoscale resolution. Furthermore, the sample’s surface can be probed over a wide analytical area range (usually between 1 µm^2^ and 10^4^ µm^2^) providing insights into local variations in sample composition, as well as giving a general overview of the sample’s structure. Finally, as long as the sample’s surface is flat and conductive, no additional sample preparation is needed prior to TOF-SIMS measurements. Despite many advantages, TOF-SIMS analysis can be challenging, especially in the case of weakly ionizing elements. Furthermore, mass interference, different component polarity of complex samples, and matrix effect are the main drawbacks of this technique. This implies a strong need for developing new methods, which could help improve TOF-SIMS signal quality and facilitate data interpretation. In this review, we primarily focus on gas-assisted TOF-SIMS, which has proven to have potential for overcoming most of the aforementioned difficulties. In particular, the recently proposed use of XeF_2_ during sample bombardment with a Ga^+^ primary ion beam exhibits outstanding properties, which can lead to significant positive secondary ion yield enhancement, separation of mass interference, and inversion of secondary ion charge polarity from negative to positive. The implementation of the presented experimental protocols can be easily achieved by upgrading commonly used focused ion beam/scanning electron microscopes (FIB/SEM) with a high vacuum (HV)-compatible TOF-SIMS detector and a commercial gas injection system (GIS), making it an attractive solution for both academic centers and the industrial sectors.

## 1. Introduction

### 1.1. Principles of TOF-SIMS

Time-of-flight secondary ion mass spectrometry (TOF-SIMS) is a surface analysis technique, which allows to characterize a material’s chemical structure in 3D with nanoscale resolution. In the excellent books from Benninghoven [1], van de Heide [2], Vickermann, and Briggs [3], the reader can find extensive information on physics fundamentals, sputtering and ionization processes, instrumentation, data acquisition, and processing, as well as an overview of commercial detectors. In brief, during TOF-SIMS measurements, a sample’s surface is bombarded with a primary ion beam, and consequently a certain population of secondary species (atoms, molecules, and ions) is ejected. Those sputtered species, which carry electric charges, are guided toward a detector with an applied electric field. The ions are recognized based on the proportionality of their mass-to-charge ratio (*m/q*) to the second power of their time-of-flight (*t*), which is needed to cover a set distance (*d*). This relation results from combining the two following equations:

(1) $$E=\frac{mv^{2}}{2},$$

where *E* is energy, *m* is mass, *v* is velocity, and

(2) E=qV,

where *q* is charge, and *V* is voltage. Consequently,

(3) $$\frac{m}{q}=\frac{2V}{v^{2}}=\frac{2Vt^{2}}{d^{2}},$$

which for a given distance and applied voltage leads to:

(4) $$\frac{m}{q}\sim t^{2}.$$

This means that heavier ions arrive to the mass analyzer slower than the light ions. However, this relation also implies that ions of the same (or very similar) mass reach the detector at the same time. This phenomenon is called mass interference and can make TOF-SIMS data interpretation difficult. The most common method to recognize the presence of mass interference relies on comparing the measured isotope abundance to the natural isotope abundance [4,5,6]. However, this method does not apply to monoisotopic elements, such as Be, F, Na, Al, P, Sc, Mn, Co, As, Y, Nb, Rh, I, Cs, Pr, Tb, Ho, Tm, Au, Bi, and Pa. A detailed discussion on mass interference types (such as isobaric mass interference, mass interference with hydrides, oxides, hydroxides, and hydrocarbons) can be found in refs. [6] and [7].

In the secondary ion yield [2], (*Y_I_*) is defined as the ratio of the number of generated ions and (*N_IS_*) to the value of the sputtered population (*N_PS_*):

(5) $$Y_{I}=\frac{N_{IS}}{N_{PS}}$$

Note that in some books, the ion yield is referred to as the number of secondary ions per incident primary ion [8,9]. Usually, not all ions generated during a sputtering event can be detected. Therefore, for practical applications, the useful ion yield (*Y_IU_*) is considered:

(6) $$Y_{IU}=\frac{N_{ID}}{N_{AS}}$$

where *N_ID_* denotes the number of detected ions, and *N_AS_* is the number of sputtered atoms. *Y_IU_* values depend not only on the ionization efficiency but also on instrumental parameters and detector type [10,11,12].

The ionization process strongly depends on element type (ionization energy and electron affinity in the case of positive and negative ions, respectively) and surface chemical state (the so-called matrix effect [1,2,6,13,14,15,16,17]). The latter means that the efficiency of generating secondary ions is strongly affected by the type of neighboring atoms/molecules [14,18], the type of primary ion beam [12,18,19,20] and the presence of additional gases [5,6,21,22,23,24] delivered to the analytical chamber. The matrix effect can cause the number of generated ions to vary by five orders of magnitude or more, making quantification extremely difficult or even impossible. Several studies have shown that using MCs^+^ and MCs^2+^ ions (instead of M^+^ ions directly) composed of an ion of interest (M) and Cs originating from an analysis primary ion beam can help reduce the matrix effect [25,26]. Promising results were also obtained when using MA^+^ ions, where A denotes alkali primary ions, such as K^+^, Rb^+^, and Na^+^. In the case of organic materials on silicon and polypropylene substrates, metal-assisted SIMS (MetA-SIMS, i.e., depositing an Ag layer) prior to TOF-SIMS measurements have also exhibited potential for mitigating matrix effects [27]. Similar effects were observed using a water cluster beam for the analysis of amino acids combined with trehalose [28]. Finally, the surface chemistry can be de-convoluted [29] from the matrix effect during data processing with dedicated algorithms, such as PCA (principal component analysis) [30,31,32].

Topography effects [33,34,35,36] are another important aspect to consider when planning TOF-SIMS experimental chemical data and analysis. Sample geometry (3D objects, such as wires, fibers, pillars, cylindrical samples, or the presence of cracks/holes in the sample), as well as the sample’s spatial orientation can cause unwanted distortion of the extraction field, and consequently, modify the secondary ion signal. This can hinder accessing chemical information and/or lead to data misinterpretation. Recommendations on how to minimize topography effects during TOF-SIMS on conductive and insulating samples can be found in ref. [34] and ref. [35], respectively. Furthermore, the implementation of dedicated experimental setups (in micro- or macroscale [36,37]) can help to improve ion extraction in the case of relatively large samples. Furthermore, topography effects can be corrected by combining chemical data (acquired with TOF-SIMS) with topographical data obtained via ex-situ techniques, such as atomic force microscopy (AFM), confocal microscopy (CM), and digital holographic microscopy (DHM) [38,39,40,41]. The applicability of this approach can be extended by additionally including an empirical sputter model [42].

The discussed TOF-SIMS principles and properties ultimately determine the technique’s functionality and the application scope. The advantages and disadvantages of TOF-SIMS are summarized in Table 1.

### 1.2. Data Acquisition, Processing, and Representation

During a TOF-SIMS measurement, a primary ion beam scans the chosen area pixel by pixel, line by line, and frame by frame (acquisition scan). Consequently, a 4D dataset (*x*, *y,* and *z* coordinates and a corresponding mass spectrum for each data point) is obtained, providing parallel detection of all ions (assuming a sufficiently high ionization efficiency) within the previously specified mass range. Since dynamic SIMS is a destructive technique, a crater is milled into the sample’s surface. The lateral dimensions are specified by a user, and the crater’s depth depends on the material’s sputtering rates at given primary ion beam parameters (type of ions, energy, and current). By default, the *z*-dimension in TOF-SIMS data is given as a number of acquisition scans or sputtering time. The conversion to length units (in nanometers or micrometers) usually cannot be directly performed using only the TOF-SIMS data. This is only possible when the sputter rates are known, which very often is not the case for new/model materials measured under optimized experimental conditions. This can be achieved using in situ or ex situ AFM [43,44] or CM measurements. Importantly, during the sputtering process, material re-deposition can occur close to crater edges. Therefore, to prevent the appearance of potential artifacts, it is usually advised to exclude the margins from data analysis. Commonly, the 25% rule is used, e.g., in the case of a 10 µm × 10 µm scan area, only the central 5 µm × 5 µm is considered.

As was previously mentioned, a certain measured time-of-flight corresponding to a given *m/q* can represent more than one ion (mass interference). Therefore, initial knowledge of the sample’s composition (as well as any potential source of contamination during sample fabrication and processing) and experimental conditions (the type of primary ions and supplementary gases, potential contaminations of the analytical chamber from previous experiments) is required. The first step of TOF-SIMS data analysis is mass calibration of a mass spectrum, which needs at least two sufficiently high peaks. Usually, more accurate mass calibration is achieved when more peaks are taken into account. Furthermore, it is recommended to use at least one peak corresponding to a light and heavy element each. Frequently, mass calibration is performed using the signals of the sample’s main isotopes and the isotopes of the primary ion beam.

Moreover, since the electric field is applied to guide ions toward the mass analyzer, only positive or negative ions can be detected from a given analytical volume. In conjunction with the destructive nature of TOF-SIMS, this implies that it is not always possible to directly obtain complete chemical information on a sample’s structure. This occurs, for example, in the case of complex structures, whose components ionize either positively or negatively. In general, most metals ionize with a positive charge (exceptions include Au, Pt, and Sb; see Section 2.4) and typical contaminants (such as C, N, O, and F) ionize with a negative charge. Therefore, to correlate the signals of sample components, often complex molecules are considered. For example, metal oxides are used to represent the distributions of metals in the negative ion detection mode. However, this solution is not always representative [4,45].

**Figure 1:**
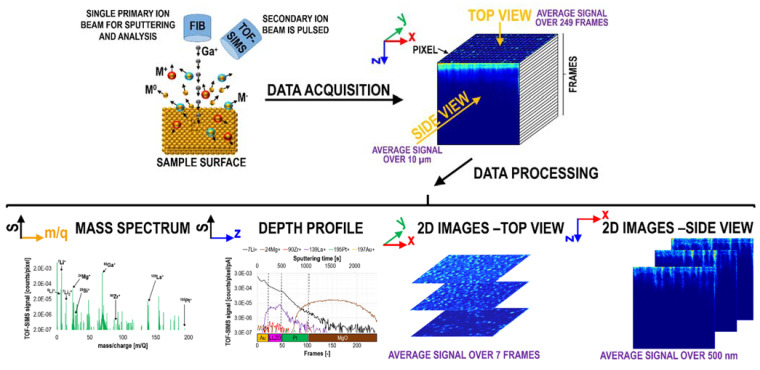
TOF-SIMS data acquisition and representation. Reprinted with permission from A. Priebe et al., “Detection of Au^+^ Ions During Fluorine Gas-Assisted Time-of-Flight Secondary Ion Mass Spectrometry (TOF-SIMS) for the Complete Elemental Characterization of Microbatteries”, ACS Applied Materials & Interfaces 2021 13 (34), 41262–41274 [22]. Copyright © 2021 American Chemical Society.

TOF-SIMS data can be represented in various ways (Figure 1), depending on the data quality and the desired level of complexity. The most general and important data are mass spectra, where the signal is integrated over the entire (measured or specified by a user) volume of interest and shown as a function of *m/q* (or sputtering time). Mass spectra are most commonly used to verify the presence of a given element in a sample (due to the very high TOF-SIMS sensitivity) and to recognize the presence of mass interference (by comparing the measured isotopic ratios to the natural abundance of an element). Depth profiles represent TOF-SIMS signals integrated in the lateral (*x-y*) plane and are given as a function of sputtering time or the number of scans/frames. They are usually used when wanting to depict a global trend across a sample, e.g., for multilayer structures they enable investigating the relative element distribution, studying processes occurring at interfaces, determining diffusion, or comparing surface structures with the buried part of a sample. Finally, 2D or 3D chemical maps can be generated to access the most detailed information (assuming sufficiently high count rates) of the sample’s chemical structure. This is particularly important in the case of complex inhomogeneous structures, such as nanoparticle-containing systems, hybrid materials, samples exposed to corrosive solutions, etc.

### 1.3. Focused Ion Beam Time-of-Flight Secondary Ion Mass Spectrometry (FIB-TOF-SIMS)

In this review, we primarily focus on the advances in the field of high vacuum (HV)-compatible TOF-detectors (CTOF—compact TOF and HTOF—high mass resolving power TOF) [46,47,48], which can be integrated within commercial FIB/SEM (focused ion beam/scanning electron microscope) instruments. Both detector types are based on orthogonal TOF (o-TOF) [49], meaning that the secondary ion beam is pulsed in the orthogonal direction with respect to the initial flight path of generated ions. The start point of the acquisition time is set by a pulser, and the ions’ time-of-flight (determined by their *m/q*), over which they cover the distance from the pulser to the detector, is recorded. It is worth mentioning that the instrumentation setup is quite different compared to dedicated standalone TOF-SIMS instruments, which operate under ultra-high vacuum (UHV) conditions. The comparison between CTOF/HTOF and TOF.SIMS^5^ instruments is provided in Table 2. Until now, there have been no systematic studies focused on exploring the maximum potential and drawbacks of the different types of TOF-SIMS detectors. To date, only two studies can be found that present CTOF and TOF.SIMS^5^ chemical data from the same sample, while additionally including a comparison with scanning transmission electron microscopy combined with energy-dispersive X-ray spectroscopy (STEM/EDX) can be found [20,50].

In the literature, the reader can find the comparison between FIB-TOF-SIMS detectors and quadrupole mass spectrometers [48], helium ion microscopy SIMS (HIM-SIMS) [51], and STM/EDX [20].

**Table 2:** Comparison between HV-compatible TOF-SIMS add-ons to FIB/SEM (CTOF and HTOF) and dedicated standalone TOF-SIMS detectors operating under UHV (TOF.SIMS^5^).

	CTOF and HTOF (from TOFWERK)	TOF.SIMS^5^ (from IONTOF)
Type of detector	Add-on to FIB-SEM instruments ^(a)^	Dedicated standalone detector
Primary ion beam species	Ga^+^ (potentially also Ne, He, Xe, O, and N) [6]	Ga, Bi*_n_*, O_2_, Cs, Ar, Xe, SF_5_, C_60_
Number of available primary ion beams	1 (used for sputtering and analysis)	2 (one primary ion beam for sputtering and the other for analysis)
Type of primary ion beam	Continuous	Pulsed
Vacuum	HV ^(b)^	UHV
Lateral resolution	<50 nm ^(c)^	<60 nm ^(d)^
Depth resolution	<10 nm	<1 nm
Mass resolving power	700–1100 Th/Th for CTOF 3000–7000 Th/Th for HTOF	>10,000 Th/Th
Mass resolution	fixed, cannot be easily modified	can be increased by decreasing the pulse duration [52,53,54]
Detection limit	ppm	ppm-ppb
Ion extraction optics	optimized to fit into existing FIB/SEM systems	maximized
Standard gases available for ion yield enhancement	XeF_2_ and H_2_O	O_2_ flooding

^(a)^ Compatible with FIB-SEM instruments from TESCAN, ORSAY Physics, ZEISS, and Thermo Fisher Scientific. ^(b)^ Usually, CTOF and HTOF are used under HV but a recent publication reports experiments under UHV [55]. ^(c)^ The Ga ion beam spot size can be as small as 2.5 nm at 1 pA ion current [56]; thus, the lateral resolution can potentially be much higher (see ref. [20]). ^(d)^ The highest lateral resolution of <20 nm was achieved using Bi_3_ clusters [57], and the highest depth resolution of <1 nm has been reported [58].

The functionality of HV-compatible TOF-SIMS detectors integrated within FIB-SEM instruments has been demonstrated for imaging nanoparticles embedded in an alloy matrix [50] or buried under thin films [20], studying thin films of pure metals [21,22,48,59], alloys [5,6,14,47,59,60,61,62,63,64,65,66,67,68,69,70], complex multilayer structures [5,6,24,46,71], solar cells [72,73,74,75], Li batteries [22,45,76,77,78,79,80,81,82,83,84,85,86,87,88,89,90,91,92,93], organic cathodes [94], vertical-cavity surface-emitting lasers (VCSEL) [43,46,47], photoanodes [95], doped semiconductors [96], complex nanotube-based systems [97], complex nanostructures [98], chemiresistors [99], geological materials [100,101,102,103,104,105,106,107,108,109,110,111,112,113], biological samples [114,115], tribofilms [116], dopants in optical fibers [117], black phosphorus [118], historical paintings [119], heterojunctions [120], ceramics [121,122], and solid oxide fuel cells (SOFC) [123,124,125,126]. Furthermore, it has been recently demonstrated that a TOF detector integrated within a FIB-SEM instrument can be used for focused-electron-beam-induced mass spectrometry (FEBiMS) to study the fragmentation of electron-sensitive materials [127].

Finally, since the CTOF and HTOF are compatible with FIB/SEM, this gives a great opportunity for conducting complementary studies in situ (without breaking vacuum conditions). Consequently, the sample’s chemical structure measured with TOF-SIMS can be compared to the data obtained using EDX and WDS (wavelength-dispersive X-ray spectroscopy) and can be further correlated with its nano- and microstructure/topography (using SEM), crystallographic structure (using electron backscatter diffraction, EBSD), topography and mechanical properties (with AFM), optical information (cathodoluminescence, CL), and vibration modes (Raman spectroscopy).

### 1.4. Gas Injection System (GIS)

**Figure 2:**
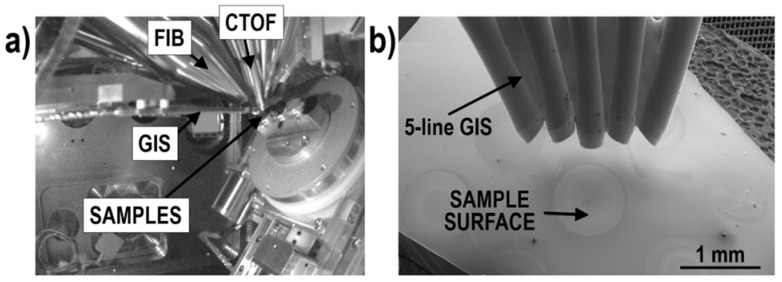
Interior of a FIB/SEM analytical chamber (**a**) and a SEM image of a 5-line GIS (**b**).

A gas injection system (GIS, Figure 2) [128,129,130] is a well-known and frequently used tool for etching and gas-assisted deposition via FIB [131] and FEB [132] (focused electron beam). It consists of a reservoir (which stores solid, liquid, or gaseous precursors), supply system, and nozzle. The precursor molecules are dissociated by the applied beam (FIB or FEB) offering a wide range of applications. Commonly, GIS is used during sample lift-out (for depositing protective layers and contacts between a sample and a micromanipulator) for X-ray computed nanotomography experiments [36,133,134], TEM lamella preparation [135], and tips for atom probe tomography (APT) [136,137]. Furthermore, the deposited layers can help decrease charging effects during FIB milling. Gases delivered via GIS can increase sputter rates [138,139], hamper FIB-induced artifacts (e.g., curtain effect [131,140]), and reduce material re-deposition [141]. Finally, GIS can be used for direct writing of 3D functional nanodevices [142,143,144,145].

For practical reasons, the local pressure at a sample’s surface can be simulated using the Empa freeware GIS simulator [146,147,148]. This software can also provide indications on the preferential orientation of a gas nozzle in the vacuum chamber to maximize/optimize gas fluxes. Note that in some cases, the insertion of a GIS can modify secondary ion extraction during FIB-TOF-SIMS.

## 2. Advances in Gas-Assisted FIB-TOF-SIMS

This review is dedicated to the recent advances in gas-assisted FIB-TOF-SIMS. The pioneer systematic studies have been conducted at Empa on multiple model samples of various complexities (i.e., beginning with pure metals and alloys, and finally covering complex metal–ceramic thin films, multilayers, and real-life samples). The main motivation of these efforts was to overcome typical drawbacks of TOF-SIMS as well as to provide solutions for enhancing the functionality and, in turn, the application scope of HV-compatible TOF-SIMS add-ons to FIB/SEMs (whose ion extraction optics are not maximized, as in the case of dedicated standalone instruments; however, it is optimized to fit into existing FIB/SEM analytical chambers). In most cases (apart from the home-built Cs evaporator), these objectives were achieved using a commercially available GIS, which was compatible with FIB-TOF-SIMS. The developed protocols allow for upgrading FIB/SEMs into advanced chemical analysis stations at relatively low costs, making them accessible to a wide group of users, both from academic centers and industrial sectors.

### 2.1. Enhancement of Negative Secondary Ions with a Home-Built CS Evaporator

Cesium has the lowest (1.8 eV) work function (minimum energy needed for releasing an electron from a surface) and the highest electropositivity (the ability to form a positive ion [149,150]) among all elements in the periodic table. Consequently, Cs can significantly decrease the work function of many metals and semiconductors [151,152,153,154,155] and can be used for increasing secondary ion yields [2,18,156,157,158,159,160]. This property was broadly investigated and successfully implemented using different methodologies, such as Cs/Xe co-sputtering [3,161,162,163], combining Cs^+^ sputtering beam with Ga^+^ [164,165], Bi^+^, or Bi_3_^+^ [25,26,166] analysis beams in dedicated dual-beam systems, coating sample surfaces with Cs prior to TOF-SIMS measurements [167], and using a special column delivering a collimated stream of neutral Cs [159,168,169,170].

**Figure 3:**
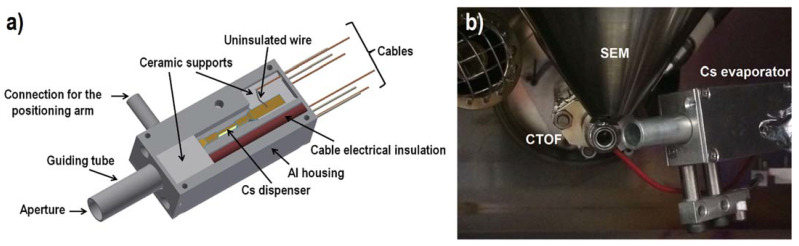
Gas-assisted FIB-TOF-SIMS with a homemade instrumentation: (**a**) Schematic of a Cs evaporator and (**b**) spatial arrangement of the experimental setup in a FIB/SEM analytical chamber (FIB column is located behind the SEM; therefore, it is not visible here) [23]. Reprinted from Ultramicroscopy, Vol 196, A. Priebe and J. Michler, Application of a novel compact Cs evaporator prototype for enhancing negative ion yields during FIB-TOF-SIMS analysis in high vacuum, Pages 10–17 [23], Copyright © 2019, with permission from Elsevier.

**Figure 4:**
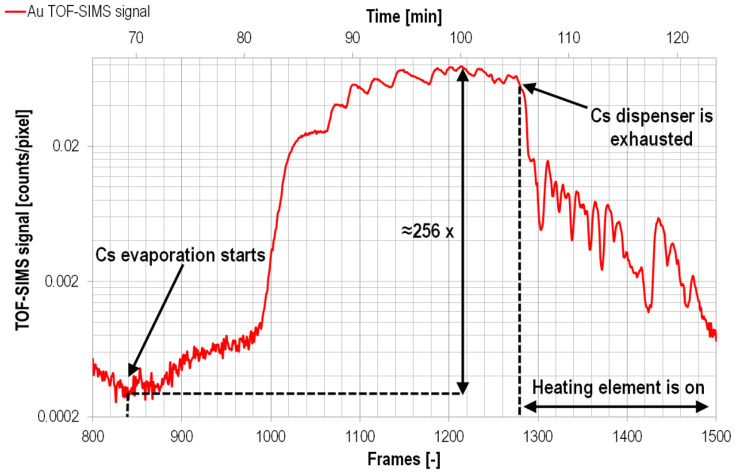
Cs-induced enhancement of the Au^−^ secondary ion signal by two orders of magnitude, measured via FIB-TOF-SIMS [23]. Reprinted from Ultramicroscopy, Vol 196, A. Priebe and J. Michler, Application of a novel compact Cs evaporator prototype for enhancing negative ion yields during FIB-TOF-SIMS analysis in high vacuum, Pages 10–17 [23], Copyright © 2019, with permission from Elsevier.

However, all of these approaches demanded either expensive dedicated instrumentation and/or operating under UHV conditions. Furthermore, these solutions cannot be easily adapted in the case of HV-compatible TOF-SIMS add-ons integrated within standard FIB/SEM systems, whose application scope is preliminary focused on sample preparation and electron-based sample characterization (e.g., EBDS or EDX). Therefore, to enable Cs atom delivery to a sample’s surface, a novel compact laboratory Cs evaporator prototype was designed and fabricated [23]. Its operation (Figure 3) is based on applying commercially available solid alkali metal dispensers (AMDs). Since AMDs are made of stable Cs salts [171], they are safe, which is a great advantage over highly reactive Cs metal. By applying an electric current to the dispenser, which induces Joule heating, neutral Cs is evaporated. The conducted TOF-SIMS experiments [23] showed that using the described setup can increase the Au^-^ ion signal by at least a factor of 256 (Figure 4) compared to experiments conducted without any supplementary gases. Moreover, experimental condition optimization (i.e., the electric current, temperature, and use of multiple Cs dispensers) can ensure a stable Cs flux for a sufficiently long time (almost 1.5 h) to conduct TOF-SIMS depth profiling (requiring usually tens of minutes) of complex samples, such as heterogeneous structures and thin film-based multilayers. However, the main drawback of using Cs dispensers is the long waiting time needed to reach the thermal equilibrium (between 1.5 h and 4.5 h, depending on the experimental settings).

### 2.2. Enhancement of Positive Secondary Ions with H_2_O and XeF_2_

The functionality and potential application scope of the Cs evaporator was demonstrated for increasing the generation of negative secondary ions during TOF-SIMS measurements. However, the adaptation of the described experimental setup to enhance positive ion yields was not possible, as available on-the-market commercial AMDs, besides Cs, offer only Na, K, Rb, and Li. Neither oxygen nor water molecules, which are known for increasing positive secondary ion yields, could therefore be delivered to a sample’s surface. Therefore, method development was focused on finding well-set commercially available solutions, which could be potentially adapted for the needs of FIB-TOF-SIMS.

As mentioned in the Introduction, the combination of FIB/SEM with GIS is broadly used during sample preparation. Furthermore, available GIS can deliver more than one gas precursor on a regular basis and provide the opportunity of testing various non-standard gas precursors (however, this can be at the cost of losing the instrument’s warranty). These made GIS an excellent candidate for FIB-TOF-SIMS applications.

Gas-assisted FIB-TOF-SIMS measurements were conducted with a standard commercial 5-line GIS. Among various possible gas precursors, MgSO_4_·7H_2_O and XeF_2_ were assigned as the most likely agents for modifying positive secondary ion generation, as the primary ion beam-induced defragmentation of the gas precursor molecules was expected to provide H_2_O (or O) and F, respectively, in the vicinity of the sample’s surface. The functionality of employing GIS for improving the chemical characterization was verified using two main HV-compatible TOF-SIMS add-ons to the FIB/SEM: CTOF [5,14,21] and HTOF [6,22,24,59,88,89] (the experimental setup is shown in Figure 2a). Whilst the combination of CTOF and GIS allowed the nominal positions of the two instrumentations to be applied, the use of HTOF required dedicated calibration tests [6,59]. This results from the high-resolution detector (HTOF) being located much closer to the sample’s surface compared to the compact one (CTOF) and, consequently, has different ion extraction optics. Therefore, the nominal GIS working distance usually used for enhancing sputter rates or depositing protective coatings cannot be used because the proximity of the GIS nozzle modifies the TOF extraction field lines and prevents sufficient secondary ion collection.

Ionization processes are currently still not completely understood, although over three decades have passed from the publication of the famous book on SIMS written by Benninghoven [1]. In the meantime, numerous dedicated studies have been performed to find the driving mechanisms of this phenomenon and have even led to disputes between researchers [15,16,17]. However, no officially approved ionization model so far exists that would be in agreement with both experimental data and theoretical calculations. The dependency of secondary ion generation on the surface’s chemical state (matrix effect, see Introduction) is even more complicated when additional parameters, such as supplementary gas, are added to an experimental setup. Therefore, to verify the benefits and potential hazards of combining FIB-TOF-SIMS with GIS, a series of separate dedicated studies have been conducted on many different samples with increasing chemical architecture complexity.

First, the influence of water vapor and XeF_2_ was verified on pure metals. In this review and previous publications, the term “fluorine gas” is often used as it is expected that the XeF_2_ gas precursor will undergo defragmentation by the impacting primary ion beam and effectively produce F. Fluorine plays a dominant role during TOF-SIMS due to its extreme electronegativity (*χ_F_* =3.98 in Pauling scale, the highest in the periodic table), whilst Xe is a noble gas. It has been observed that the simultaneous co-injection of a supplementary gas can significantly modify ionization and sputtering processes. Furthermore, the response to the presence of a supplementary gas is characteristic for a given element, its type, and the amount of the delivered gas [21]. Enhanced secondary ion production, resulting in improved signal-to-noise ratios and, in turn, lower statistical fluctuations, was expected to increase the spatial resolution and ultimately improve the quality of 2D and 3D chemical images. Additionally, it has been possible to exclude concerns related to potential preferential sample sputtering and the degradation of sample isotope abundance (resulting from introducing additional elements to a system, which could result in new mass interference scenarios), based on results obtained for different two-element Zr-based alloys [66]. Furthermore, it was observed that an alloying element can completely invert the delivered gas’s effect on the ionization efficiency of the element of interest. Finally, fluorine gas-induced modifications of metal–oxygen interactions allowed for lower (compared to the experiments without any gas) TOF-SIMS signal variations to be obtained in the proximity of sample boundaries, providing more representative data with respect to the sample’s composition.

In Table 3, a summary of all currently available information on the effect of co-injecting supplementary gases (with GIS) on the ionization efficiency and sputtering processes during FIB-TOF-SIMS conducted with a Ga^+^ primary ion beam under HV conditions is presented. The gas enhancement factor for the TOF-SIMS signal (*GAE_TOF-SIMS,gas_*) of a given element (*M*) is defined as a ratio of the total signal measured during gas-assisted measurement (*S_gas_*) to the total signal measured without any gas (*S_ref_*), i.e., at background pressure from the same volume (note that TOF-SIMS is a destructive technique; therefore, two measurements cannot be acquired from exactly the same location):

(7) $$GAE_{TOF-SIMS,gas}\left(M\right)=\frac{S_{gas}\left(M\right)}{S_{ref}\left(M\right)},$$

The value of *GAE_TOF-SIMS,gas_* is also equivalent to the enhancement of an element’s useful yields (see Equation (6)).

Another parameter, which helps determine a gas’s effect during FIB-TOF-SIMS, is the gas enhancement factor for sputter rates (*GAE_SP_*):

(8) $$GAE_{SP}\left(M,gas\right)=\frac{Y_{SP,gas}\left(M\right)}{Y_{SP,ref}\left(M\right)},$$

Since the sputter rate (*Y_SP_*) defines the number of sputtered atoms from the target per unit of time [129] and the compared volumes are identical in the case of studying thin films (for bulk materials, additional instrumentation, such as SEM, AFM or CM, is needed to assess the sputter depth), the *GAE_SP_* can be calculated based on depth profiles:

(9) $$GAE_{SP}\left(M,gas\right)=\frac{t_{int,ref}\left(M\right)}{t_{ref,gas}\left(M\right)},$$

where *t_int_* is the time needed to reach an interface between the sample and the substrate.

Note that the values of *GAE_TOF-SIMS_* and *GAE_SP_* are not only functions of studied material and used gas but also depend on the primary ion beam. However, to date, gas-assisted FIB-TOF-SIMS measurements under HV have been only conducted using a Ga^+^ primary ion beam (for both sputtering and analysis), and no comparison with other species has been provided.

In can be seen in Table 3 that fluorine gas has a tremendous effect on the ionization efficiency as it can enhance the generation of positive secondary ions by up to two to three orders of magnitude. Simultaneously, however, it hampers the production of negative secondary ions. This means that XeF_2_ should not be used for experiments conducted in the negative ion detection mode. Remarkably, in the case of elements that dominantly ionize with a negative charge under standard vacuum conditions (i.e., without any gas), such as Au or Pt, positive secondary ions are observed during fluorine gas-assisted FIB-TOF-SIMS. The observed charge inversion is discussed in the following section of this review.

### 2.3. Fluorine Gas-Induced Enhancement of Spatial Resolution and Separation of Mass Interference

As previously stated, in the case of TOF-SIMS, three main parameters, i.e., mass resolution and lateral and depth resolution, have to be optimized to achieve the desired quality of chemical data. The primary ion beam, i.e., the type of ions, beam energy, and beam current, determine the spatial resolution. Under standard vacuum conditions, the highest lateral resolution is obtained when using the highest possible primary beam energy (usually 30 keV, in the case of a Ga^+^ primary ion beam), whilst the highest depth resolution requires using low primary ion beam energies (usually ≤ 5 keV) [5,131]. This implies that either two separate measurements have to be conducted under different experimental conditions from two different sample volumes to assess the highest lateral resolution and the highest depth resolution, or the experimental conditions are compromised to obtain the best possible (but not maximized) lateral resolution and depth resolution from the same analytical volume (in particular when 3D representation of the sample’s chemical structure is the ultimate objective). Furthermore, adjusting the experimental conditions at low primary ion beam energies (i.e., five parameters, including FIB focus, *x*- and *y*-stigmators, *x*- and *y*-centering) is usually challenging and time-consuming, as the effects of the applied parameter’s changes cannot be directly determined via FIB (as in this case, the lateral resolution is low), and additional imaging using SEM is required. The step-by-step methodology of setting the beam parameters is illustrated in ref. [5] in the Supporting Information. In that work, the influence of different primary ion beam energies and currents was shown when depth profiling a model Cu/Zr/ZrCuAg@Si multilayer. Among various configurations, the optimal results were achieved using a 5 keV ion beam energy and 50 pA ion current (i.e., low beam energy and low beam current), which allowed to assess all interface locations between the subsequent thin films. However, a strong mass interference at *m/q* = 107, corresponding to ^107^Ag^+^ and ^91^Zr^16^O^+^ ions prevented obtaining a correct Ag distribution (using the main element’s isotope) across the multilayer. Therefore, the tedious and lengthy adjustment of the primary ion beam, providing high depth resolution but low lateral resolution, which in conjunction with strong mass interference, prevented to accurately and completely characterize the sample.

**Figure 5:**
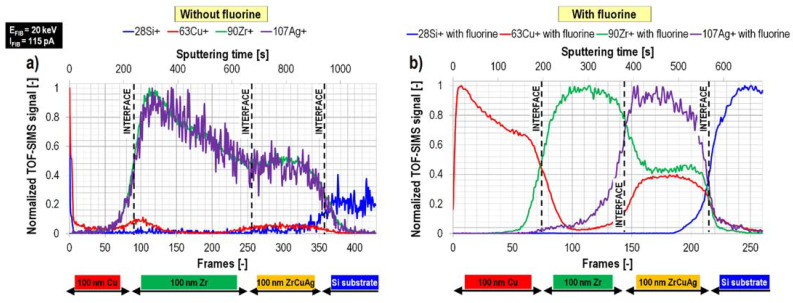
Fluorine gas-induced separation of mass interference during a FIB-TOF-SIMS measurements [5]. The TOF-SIMS depth profiles of the Cu/Zr/ZrCuAg@Si sample acquired without (**a**) and with fluorine gas (**b**). Reprinted with permission from A. Priebe et al., “Application of a Gas-Injection System during the FIB-TOF-SIMS Analysis—Influence of Water Vapor and Fluorine Gas on Secondary Ion Signals and Sputtering Rates”, Analytical Chemistry 2019 91 (18), 11712–11722 [21]. Copyright © 2019 American Chemical Society.

**Figure 6:**
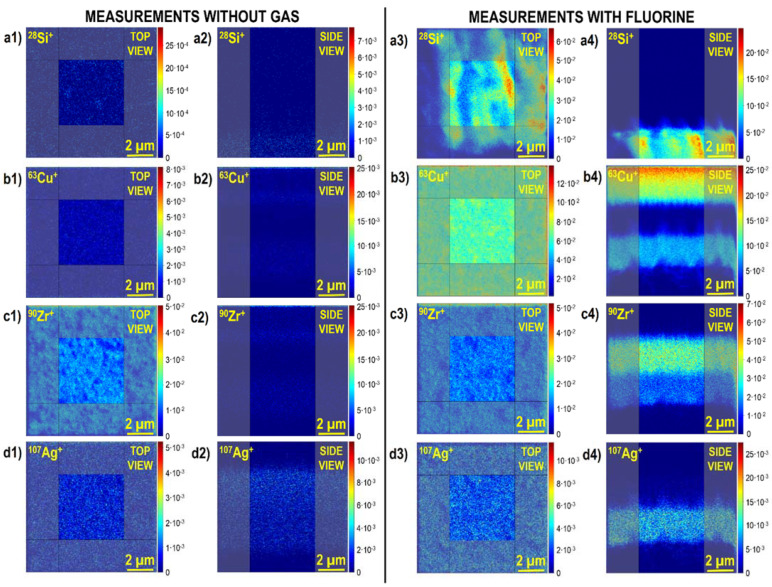
The 2D chemical images of the Cu/Zr/ZrCuAg@Si sample obtained without (**a1**–**d1**,**a2**–**d2**) and with (**a3**–**d3**, **a4**–**d4**) fluorine gas [5]. The top views (**a1**–**d1**,**a3**–**d3**) represent lateral images in the *x-y* plane, and the side views represent the depth images in the *x-z* plane. The shadowed regions show the data excluded when generating the depth profiles (given in Figure 5). Reprinted with permission from A. Priebe et al., “Application of a Gas-Injection System during the FIB-TOF-SIMS Analysis—Influence of Water Vapor and Fluorine Gas on Secondary Ion Signals and Sputtering Rates”, Analytical Chemistry 2019 91 (18), 11712–11722 [21]. Copyright © 2019 American Chemical Society.

However, subsequent experiments at intermittent primary ion beam energies (20 keV) and higher primary ion beam currents (115 pA), which in practice are much easier and faster to conduct, allowed to obtain both high lateral resolution and high depth resolution in the presence of fluorine gas at the sample’s surface. Remarkably, fluorine gas-induced separation of mass interference was observed for the first time (Figure 5). This was a significant achievement as mass interference in general is one of the major drawbacks of TOF-SIMS (see Table 1). In this case, the secondary ion signals of the sample’s main elements were enhanced by up to three orders of magnitude, resulting in statistically better data (higher signal-to-noise ratio and reduction in signal fluctuations). Consequently, improved quality 2D chemical images (both in *x-y* and *x-z* planes) were obtained, and the measured signal distributions were more representative with respect to the sample’s composition compared to the results obtained without any gas (Figure 6).

**Figure 7:**
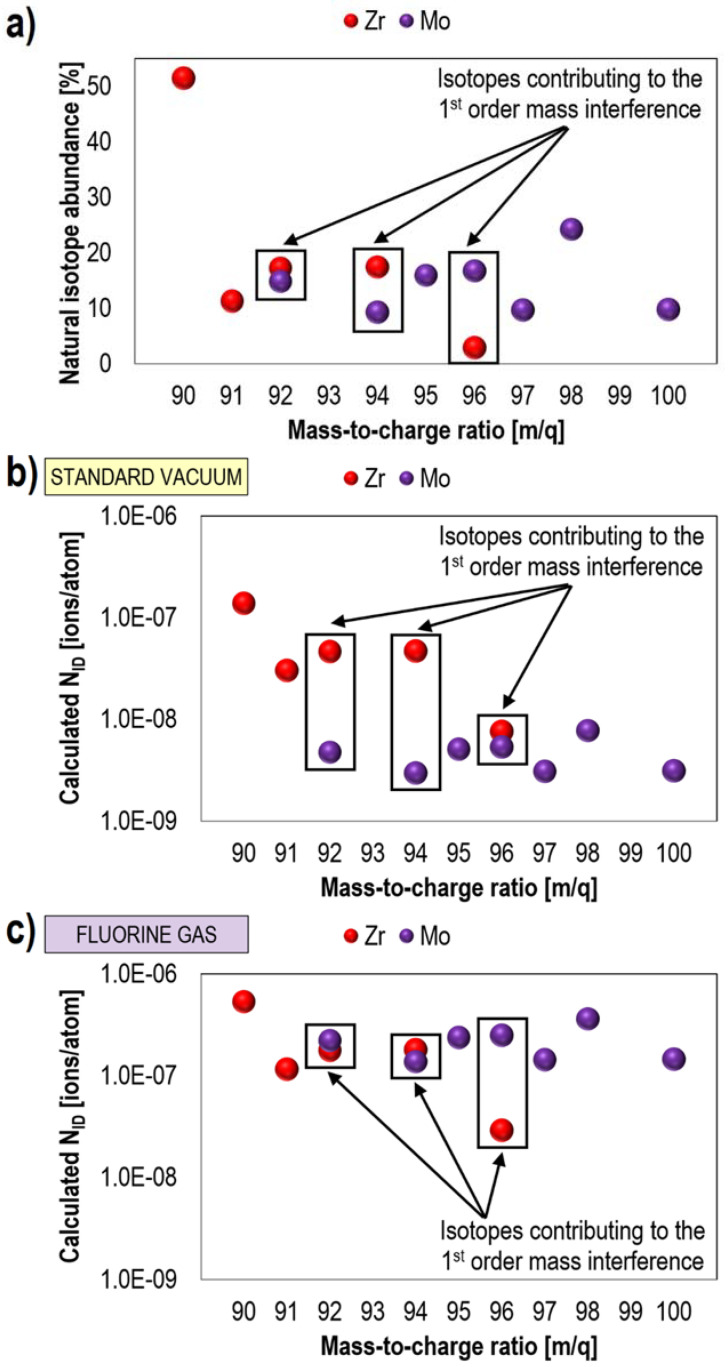
Isobaric mass interference. Comparison of Zr (red dots) and Mo (violet dots) natural isotope abundance (**a**) and number of detected ions (*N_ID_*) at different mass-to-charge ratios (*m/q*), which were calculated based on the measured TOF-SIMS signals without (**b**) and with (**c**) fluorine gas. Reprinted with permission from A. Priebe et al., “Mechanisms of Fluorine-Induced Separation of Mass Interference during TOF-SIMS Analysis”, Analytical Chemistry 2021 93 (29), 10261–10271 [6]. Copyright © 2021 American Chemical Society.

The outstanding phenomenon of gas-induced separation had never been reported before, neither for fluorine nor for any other gas. Therefore, dedicated comprehensive studies were conducted to understand its underlying mechanisms [6]. Zr, Mo, and Ag were chosen as the most representative candidates, as their combination within a specimen ensured an extreme case of multi-scenario mass interference. This included mass interference between single isotopes (i.e., isobaric interference, first-order mass interference) of Zr and Mo, whose three isotopes have almost the same *m/q* values (at *m/q* = 92, 94, and 96) and higher order mass interference, in which single isotopes, as well as complex molecules (usually hydrides, oxides, and hydroxides) take part. It was confirmed that fluorine significantly modifies the ionization process (i.e., matrix effect) during Ga^+^ primary ion beam bombardment, and the response of different elements to the presence of F is characteristic, resulting in signal variations by several orders of magnitude. Furthermore, it was observed that not only the values of *GAE_TOF-SIMS_* but also a percentage contribution of the element’s isotopes (i.e., natural abundance, Figure 7) determine the success of fluorine gas-induced separation of mass interference. Finally, it was found that fluorine affects the efficiency of generating chemical bonds between metals and H^−^, O^−^, and OH^−^. This property, for example, can be correlated with fluorine electron affinity (322 kJ/mol), which is higher than the electron affinity of oxygen (141 kJ/mol) [172] and the aforementioned extreme electronegativity of fluorine.

**Figure 8:**
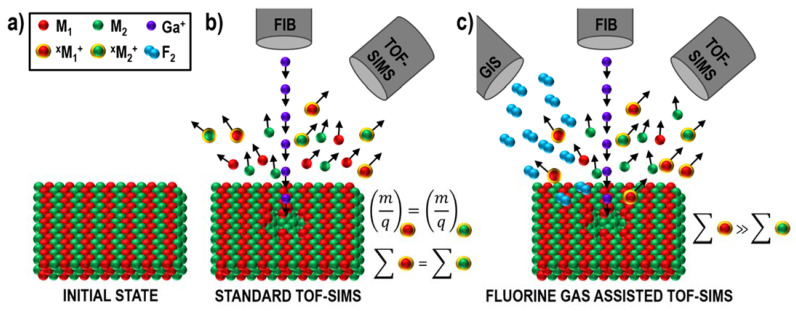
Diagram representing one of the potential mechanisms of fluorine gas-induced separation of mass interference: Change in secondary ion yield in the presence of fluorine is different and characteristic for various elements [6]. (**a**) A hypothetical sample is made of two elements *M_1_* and *M_2_*, whose neutral atoms are shown as red and green dots, respectively. (**b**) Due to Ga^+^ primary ion beam bombardment, the population of secondary species is ejected from the sample’s surface (dots with yellow halos denote ions). (**c**) A characteristic response of an element to the presence of fluorine results in different ionization efficiencies and, therefore, a different number of produced secondary ions (*∑*) of the two elements/isotopes. This can vary by several orders of magnitude. Reprinted with permission from A. Priebe et al., “Mechanisms of Fluorine-Induced Separation of Mass Interference during TOF-SIMS Analysis”, Analytical Chemistry 2021 93 (29), 10261–10271 [6]. Copyright © 2021 American Chemical Society.

**Figure 9:**
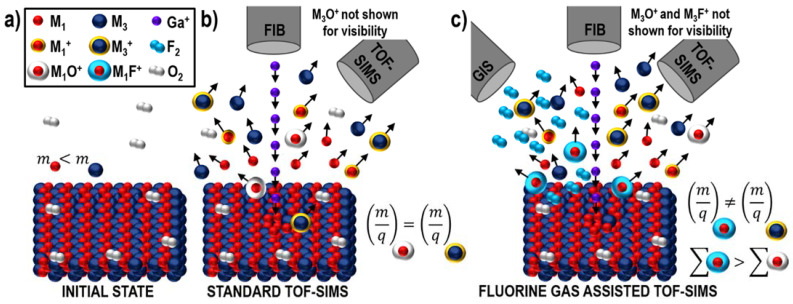
Diagram representing one of the potential mechanisms of fluorine gas-induced separation of mass interference: Change in complex-ion (such as oxides or hydrides) formation efficiency [6]. (**a**) A sample is made of two elements *M_1_* and *M_2_*, whose masses are different (*m*_1_ < *m*_2_). (**b**) During Ga^+^ primary ion beam bombardment, complex ions are produced (oxygen comes from the oxidized sample surface or residual gas in an analytical chamber). (**c**) Fluorine affects the efficiency of metal–oxygen bond production, and metal fluorides are generated more favorably than oxides. Reprinted with permission from A. Priebe et al., “Mechanisms of Fluorine-Induced Separation of Mass Interference during TOF-SIMS Analysis”, Analytical Chemistry 2021 93 (29), 10261–10271 [6]. Copyright © 2021 American Chemical Society.

The obtained data indicate the two postulated mechanisms of fluorine gas-assisted separation of mass interference (Figure 8 and Figure 9) that occur simultaneously and inseparably. However, at the current stage of knowledge, it has not been possible to determine whether the contribution of any process is dominant.

### 2.4. Fluorine Gas-Induced Inversion of Charge Polarity from Negative to Positive

TOF-SIMS is a destructive technique, and under standard vacuum conditions, the experimental parameters (primary ion beam energy and current) have to be compromised if sufficiently high lateral and depth resolution are desired to characterize a given analytical volume. Furthermore, during a single measurement, only positive or negative ions can be guided by the applied electric field toward a mass analyzer and be detected. This implies that often complete chemical information cannot be assessed directly from exactly the same volume. A common method to indirectly represent the distribution of an element (or verify its presence in a mass spectrum), which ionizes with a different polarity than the rest of the sample’s components, is based on using its complex ion distributions. For example, when a sample is comprised of elements ionizing with opposite charges (i.e., most metals ionize positively and typical organic components/contaminants ionize with a negative charge), chemical characterization is conducted in the negative ion detection mode, and the metal oxide ions are used to represent the distributions of metals in the sample. However, various studies have shown that such an approach is not always representative or accurate, as metal oxide ion signals do not always follow the same pattern as metal ion signals [4,45].

The inability in obtaining complete sample chemical structure information via direct measurements is particularly problematic in the case of complex specimens, such as next generation batteries, which demand detailed knowledge on all the sample’s components and potential contaminants to understand/explain the electrochemical properties and degradation processes. Therefore, dedicated studies [22] were conducted to verify whether simultaneous co-injection of supplementary gases during FIB-TOF-SIMS can help in this matter.

Au was chosen as the most representative element as it is one of few metals, which dominantly generates negative ions during bombardment via a primary ion beam. Furthermore, due to its outstanding chemical stability, Au plays an important role in the development of rechargeable thin-film solid-state Li batteries [173,174,175,176]. Even at high temperatures, Au enables operating with extremely reactive redox species (such as Li), and thus, it can be used as a current collector. However, as a noble metal, Au is very mobile and has a tendency to diffuse during device operation [177]. This implies the necessity of representing Au distributions precisely, together with the distributions of other system components, which usually ionize with a positive charge. This is particularly important when studying processes occurring at the interfaces of multilayers (such as alloy formation or element diffusion) and to detect potential degradation factors limiting the functionality of a device.

**Figure 10:**
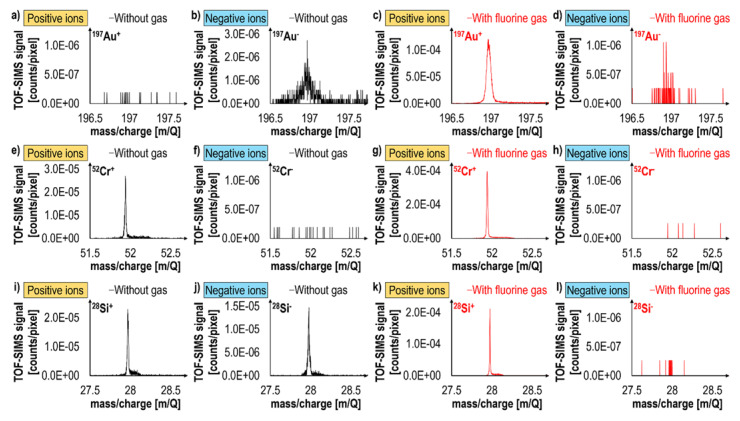
Mass spectra of a Au/Cr/SiO_2_/Si sample representing the main isotopes of Au (**a**–**d**), Cr (**e**–**h**), and Si (**i**–**l**) [22]. The data were acquired without (black lines) and with (red lines) fluorine gas in positive (yellow labels) and negative (blue labels) ion detection modes. Fluorine significantly enhances positive secondary ion generation and decreases the generation of negative secondary ions. Reprinted with permission from A. Priebe et al., “Detection of Au^+^ Ions During Fluorine Gas-Assisted Time-of-Flight Secondary Ion Mass Spectrometry (TOF-SIMS) for the Complete Elemental Characterization of Microbatteries”, ACS Applied Materials & Interfaces 2021 13 (34), 41262–41274 [22]. Copyright © 2021 American Chemical Society.

**Figure 11:**
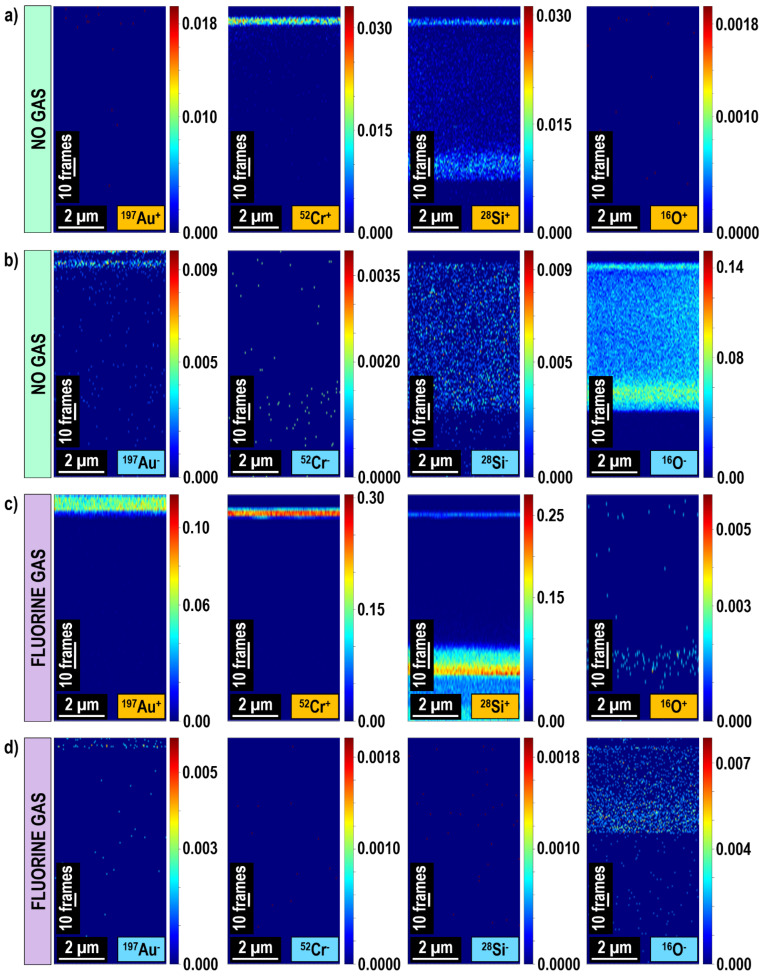
The 2D chemical maps of a Au/Cr/SiO_2_/Si sample measured without (**a**,**b**) and with (**c**, **d**) fluorine gas in positive (**a**,**c**) and negative (**b**,**d**) ion detection modes. The data are shown in depth plane (i.e., *x-z* plane). Fluorine-induced enhancement of positive ion yields and charge inversion of secondary ions, from negative to positive, allows for obtaining complete chemical information directly from the same volume during a single measurement [22]. Reprinted with permission from A. Priebe et al., “Detection of Au^+^ Ions During Fluorine Gas-Assisted Time-of-Flight Secondary Ion Mass Spectrometry (TOF-SIMS) for the Complete Elemental Characterization of Microbatteries”, ACS Applied Materials & Interfaces 2021 13 (34), 41262–41274 [22]. Copyright © 2021 American Chemical Society.

Figure 10 shows mass spectra of a Au/Cr/SiO_2_/Si sample, which were acquired during experiments conducted in the positive and negative ion detection modes as well as under standard vacuum conditions and in the presence of fluorine gas [22]. The discussed sample represents an excellent research model system as it contains elements, which under standard vacuum conditions dominantly ionize with a negative charge (Au), with a positive charge (Cr), and with both positive and negative charges (Si, a semiconductor). Extraordinarily, the addition of fluorine gas during Ga^+^ sputtering resulted not only in significant positive secondary ion signal enhancement (by an order of magnitude in the case of Cr and Si) but also in the inversion of charge polarity from negative to positive (as in the case of Au and Si). Interestingly, the Au^+^ signal measured in the presence of fluorine was two orders of magnitude higher than the dominant Au^-^ signal measured under standard vacuum conditions. Furthermore, the measured negative ion signals were decreased, i.e., roughly by a factor of three in the case of Au and to the background (noise) level in the case of Si. This study [22] proves that FIB-TOF-SIMS conducted at intermittent primary ion beam energies (20 keV) and supported with the simultaneous delivery of fluorine gas to a sample’s surface can provide enhanced resolution, direct and complete information on a sample’s 3D chemical structure, based on positive secondary ions detection (Figure 11).

This achievement is of tremendous importance for studying and developing new generation batteries, which are discussed in the following part of this review (see Section 2.6).

### 2.5. Advantages and Disadvantages of Using Fluorine during FIB-TOF-SIMS

At the time of writing this review, fluorine gas-assisted FIB-TOF-SIMS was still a new and under-development experimental approach. However, the amount of experimental data acquired on a broad spectrum of analytical specimens allowed for demonstrating its potential, proving the advantages, and observing its disadvantages or potential hazards. Table 4 summarizes all current knowledge on this subject.

### 2.6. Applications of Gas-Assisted FIB-TOF-SIMS

#### 2.6.1. Thin Films

**Figure 12:**
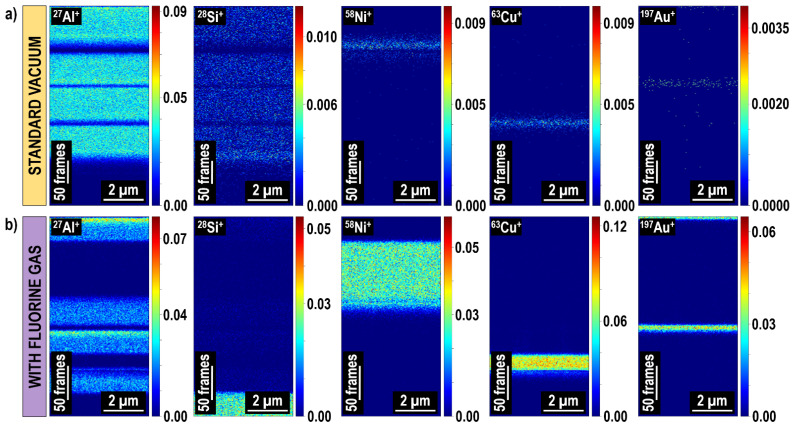
The 2D chemical maps (x-z plane) of a complex Al_2_O_3_/Ni/Al_2_O_3_/Au/Al_2_O_3_/Cu/Al_2_O_3_@Si multilayer system built of alternating ALD and PVD thin films [24]. Data were measured without (**a**) and with (**b**) fluorine gas. Reprinted with permission from A. Priebe et al., “High Sensitivity of Fluorine Gas-Assisted FIB-TOF-SIMS for Chemical Characterization of Buried Sublayers in Thin Films”, ACS Applied Materials & Interfaces 2021 13 (13), 15890–15900 [24]. Copyright © 2021 American Chemical Society.

One of the major advantages of TOF-SIMS over other analytical techniques of this type (such as STEM/EDX or APT) is a fast, efficient, and relatively easy chemical characterization of complex multilayer structures, buried thin films, and interfaces. As mentioned, TOF-SIMS does not require tedious and time-consuming lamella preparation and, as long as a sample surface is flat and conductive, no additional sample processing is needed prior to a measurement. Excellent results proving the high spatial resolution are usually demonstrated using Al signals [57,178] (note that in general Al provides very high secondary ion signals). However, the representing material’s chemical structure in 3D with nanoscale resolution in the case of weakly-ionizing elements, whose amount in a sample is very small, is still challenging. The dedicated studies [6,24] conducted on model thin films (i.e., 2D materials, whose one dimension is negligibly small compared to the two others and deposited by a progressive addition of atoms or molecules [179,180]) have shown that simultaneous co-injection of fluorine gas can dramatically increase the ionization efficiency and enable accurate representation of complex thin film-based systems. This was proved in the case of metallic–ceramic multilayer stacks whose subsequent thin film thicknesses varied even by two orders of magnitude (1–100 nm). Figure 12 shows the 2D chemical maps represented in depth (*x-z* plane) of Al_2_O_3_/Ni/Al_2_O_3_/Au/Al_2_O_3_/Cu/Al_2_O_3_@Si deposited with a novel in situ ALD-PVD (physical vapor deposition—atomic layer deposition [181]) hybrid system [182]. In the case of experiments conducted under standard vacuum conditions (i.e., without any gas, Figure 12a), although all subsequent layers can be distinguished, only Al provided sufficiently high signal. Furthermore, the mass interference at *m/q* = 28 corresponding to ^28^Si^+^ and ^27^Al^1^H^+^ prevented correct assessment of Si distribution. However, the presence of F in the analytical system (Figure 12b) enhanced the generation of all positive ion signals resulting in the higher spatial resolution and provided more representative chemical data with respect to the sample composition (i.e., no mass interference at *m/q* = 28, ^28^Si^+^ signal represents the location of Si substrate). The tremendous increase in ^197^Au^+^ signal resulting from the fluorine gas-induced inversion of charge polarity can be also observed.

**Figure 13:**
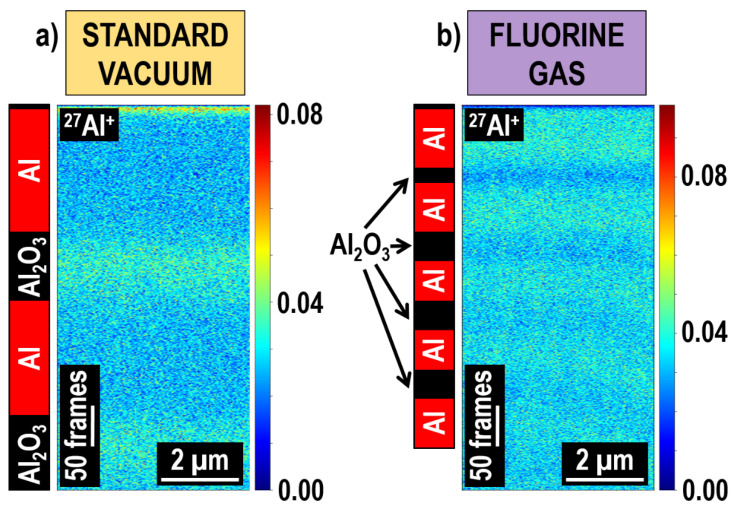
Elemental images of the Al/Al_2_O_3_/Al/Al_2_O_3_/…/Al multilayer deposited with an in situ ALD-PVD system and measured with FIB-TOF-SIMS. Due to the high sensitivity of the FIB-TOF-SIMS technique, the ^27^Al^+^ signal distribution was sufficient to assess the location of metallic PVD (Al) and ceramic ALD (Al_2_O_3_) thin films. Furthermore, higher sputtering rates during fluorine gas-assisted Ga^+^ primary ion beam milling allowed more layers to be represented (**b**) when compared to the experiments conducted under standard vacuum conditions (**a**). Reprinted with permission from A. Priebe et al., “High Sensitivity of Fluorine Gas-Assisted FIB-TOF-SIMS for Chemical Characterization of Buried Sublayers in Thin Films”, ACS Applied Materials & Interfaces 2021 13 (13), 15890–15900 [24]. Copyright © 2021 American Chemical Society.

Further studies proved the excellent potential of gas-enhanced FIB-TOF-SIMS using an extreme case of Al/Al_2_O_3_/Al/Al_2_O_3_/…/Al multilayer, whose alternating metallic and ceramic thin films varied only by the oxygen content (Figure 13). The thickness of Al layers (deposited with PVD) was around 125 nm, and the Al_2_O_3_ layers (deposited with ALD) varied from 10 to 1 nm (counting from the top) with a 1 nm decline. It was shown that even a Al_2_O_3_ thin film with thickness < 9 nm buried up to 530 nm deep below the surface (which is a lot in the case of depth profiling conducted at low primary ion beam energies) can be well and relatively fast (<1 h 15 min) distinguished from chemically similar Al thin films.

The development of as-assisted FIB-TOF-SIMS is expected to play an important role in the development and characterization of thin film-based systems (which are broadly used in microelectronics, microbatteries, solar cells, photovoltaics, as protective coatings, and many others) as their chemical composition and microstructure determine the final optical, mechanical and electrical properties, and ultimately the functionality of deposited materials.

#### 2.6.2. Li-Ion Solid State Batteries

Another field, in which gas-assisted FIB-TOF-SIMS can turn out to be unrivalled, are next generation Li-ion solid-state batteries (LIBs). These chemical systems attract a lot of attention especially in the view of global warming and the strong necessity of renewable environment-friendly energy storage systems. One of the most important applications of LIBs are electric vehicles (EVs) [183,184]. Over the last several decades, the capacity, power, and efficiency of LIBs were significantly improved [185] but the safety issues, which result from using highly flammable organic liquid electrolytes are still serious problems [186,187,188]. A great alternative to the conventional LIBs is the so-called all-solid-state batteries (ASSBs), which rely on inorganic solid-state electrolytes (SSE) [189] enabling for operation in a wide range of temperatures. The architecture of ASSBs is usually very complex as they contain buried structures and heterogeneous interfaces [186]. In conjunction with highly reactive Li, the complete characterization of such materials is very difficult. An overview of typical characterization techniques used in the battery community is provided in ref. [45]. In summary, none of the typically used methods (such as impedance spectroscopy, polarization measurements, X-ray computed nano-tomography, neutron depth profiling, magnetic resonance imagining, Raman spectroscopy, and synchrotron-based X-ray techniques) provide sufficient or any information on the sample’s chemical structure in 3D with nanoscale resolution. The latter can be achieved by three techniques TOF-SIMS, STEM/EDX, and APT, but only TOF-SIMS can provide information on light (including Li) and heavy elements and molecules constituting a sample over wide range of analytical areas. STEM/EDX cannot measure light elements (such as Li), and in this case, additional challenging electron energy loss spectroscopy (EELS) would need to be conducted [190]. Furthermore, both STEM and APT require time-consuming sample preparation (milling lamella or tips) with external instrumentation, such as FIB. Finally, the analytical volume of APT samples is limited to the uttermost part of a tip with diameter in the order of 100 nm and, therefore, might not be representative for the entire investigated material (due to potential contamination or local material segregation). Despite undeniable advantages of TOF-SIMS over STEM/EDX and APT, assessing direct and complete chemical information with TOF-SIMS can be still difficult. As mentioned, TOF-SIMS is a destructive technique and during a single measurement, either positive or negative ions can be detected. This is quite problematic in the case of Li-ion solid-state batteries as they usually contain elements, which ionize with opposite charges. However, the recently observed potential of fluorine gas-assisted FIB-TOF-SIMS for inducing a charge inversion from negative to positive allows both types of elements to be detected from the same analytical volume and consequently, the complete and direct chemical characterization to be achieved.

**Figure 14:**
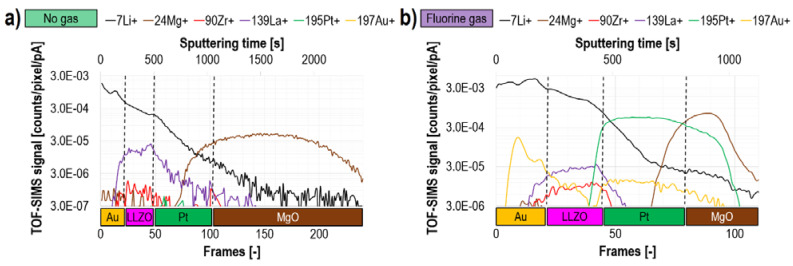
Characterization of Li-ion solid-state batteries (blocking lithium dendrite growth in solid-state batteries with an ultrathin amorphous Li-La-Zr-O solid electrolyte): Depth profiles of Au/Li7La_3_Zr_2_O_12_/Pt/MgO/Si sample. Under standard vacuum conditions (**a**), information on Au and Pt distribution is not accessible. However, simultaneous delivery of fluorine gas during Ga^+^ primary ion beam bombardment inverts the polarity of generated secondary ions from negative to positive (**b**) providing complete and direct information on the sample chemical structure. Reprinted with permission from A. Priebe et al., “Detection of Au^+^ Ions During Fluorine Gas-Assisted Time-of-Flight Secondary Ion Mass Spectrometry (TOF-SIMS) for the Complete Elemental Characterization of Microbatteries”, ACS Applied Materials & Interfaces 2021 13 (34), 41262–41274 [22]. Copyright © 2021 American Chemical Society.

A great example of the practical application of this method was presented on Au/Li_7_La_3_Zr_2_O_12_/Pt/MgO/Si [22]. The most important part of this multilayer system was a novel amorphous Ga-doped lithium garnet Li_7_La_3_Zr_2_O_12_ (aLLZO) thin film with thickness of only 70 nm. The separated dedicated studies [88] showed that aLLZO can be used as a solid electrolyte and can effectively block the growth of hazardous Li dendrites (which can short-circuit a battery) in solid-state batteries. The electrochemical measurements of Au/Li_7_La_3_Zr_2_O_12_/Pt/MgO/Si showed the difference in the background current density, which indicated the presence of an additional built-in bias in the system [22]. However, the reason for this observation could not be assessed based only on these experiments. Therefore, FIB-TOF-SIMS measurements were conducted supplementary (Figure 14) to explain the operation mechanisms and degradation processes. The TOF-SIMS data obtained during the measurements conducted without any gas (Figure 14a) were not conclusive because Au and Pt ionized with the negative charges while most of the main aLLZO components generated positive ions during Ga^+^ primary ion beam bombardment. However, simultaneous delivery of fluorine allowed all distributions of the most crucial components of the multilayer (Li, Zr, La, Pt, and Au) to be directly correlated (Figure 14b). Thus, it was possible to observe a Li-ion signal outside aLLZO and at the location of Au layer. This indicated that Li can alloy with Au, which consequently could explain the aforementioned difference in the measured current density. Remarkably, the fluorine gas-enhanced FIB-TOF-SIMS enabled the distribution of 5 ppm Au trace contamination of Pt source to be observed. This was an impressive observation taking into account a very small amount of Au and the detection of its positive ions (as under standard vacuum conditions Au dominantly generates negative ions).

#### 2.6.3. Complex Alloys

**Figure 15:**
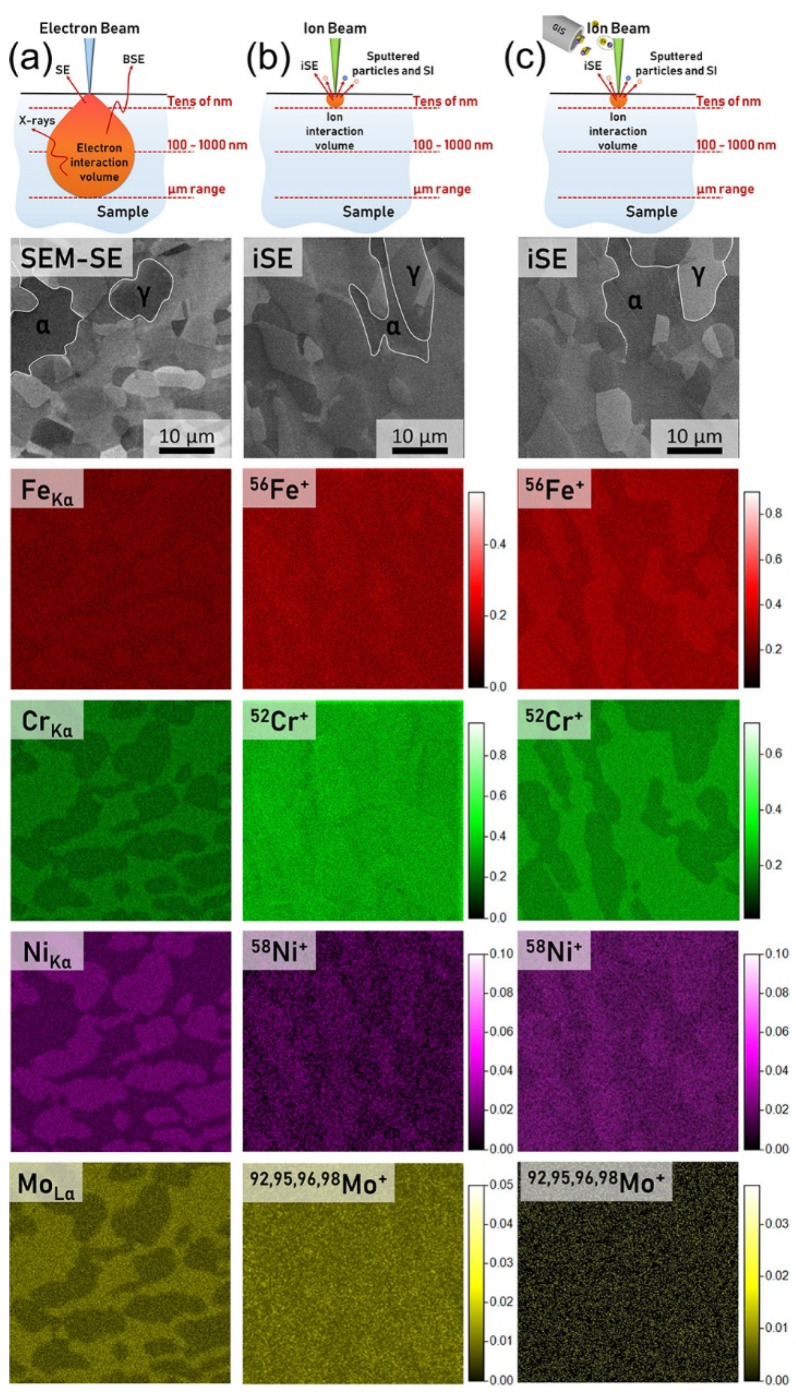
Chemical characterization of commercial 2507 super duplex stainless steel: Comparison of data obtained with EDX (**a**) FIB-TOF-SIMS under standard vacuum conditions (**b**), and during fluorine gas-assisted FIB-TOF-SIMS (**c**) [59]. Reprinted with permission from K. Wieczerzak et al., “Practical Aspects of Focused Ion Beam Time-of-Flight Secondary Ion Mass Spectrometry Analysis Enhanced by Fluorine Gas Coinjection”, Chemistry of Materials 2021 33 (5), 1581–1593 [59]. Copyright © 2021 American Chemical Society.

Another study proving the potential of gas-assisted FIB-TOF-SIMS was conducted on a commercial 2507 super duplex stainless steel [59]. This multicomponent alloy with heterogeneous microstructure was used to compare chemical data obtained using EDX, standard FIB-TOF-SIMS, and FIB-TOF-SIMS conducted with the presence of fluorine (Figure 15). The data showed that fluorine injected to the analytical chamber significantly improved the quality of lateral 2D chemical maps and allowed α and γ phases present in the steel to be precisely distinguished even though their chemical compositions were very similar. Furthermore, it was possible to obtain distinct and sharp α/γ interfaces, as during the FIB-TOF-SIMS measurements performed with Ga^+^ primary ion beam bombardment the interaction volume was much smaller than in the case of EDX.

## 3. Prospective and Future Directions

The impressive potential of combining FIB-TOF-SIMS with GIS has been demonstrated on pure metals [21], alloys [59,66], thin film-based multilayer systems [5,6,22,24], and novel batteries [22,88,89]. So far, commonly available gas precursors providing XeF_2_ and water vapor were used; however, the functionality of this solution can be extended to many other gases. Probably the most interesting and promising results could be obtained with Cs-containing gas precursors delivered during FIB-TOF-SIMS conducted in the negative ion detection mode. As previously mentioned, Cs is a well-known element for enhancing negative ion yields [2,3,23,151,152]. Furthermore, Cs is the most electropositive element in the periodic table (*χ_Cs_* = 0.79 in Pauling scale). In conjunction with the highest electronegativity of F (*χ_F_* = 3.98), one can potentially expect that the extraordinary properties of F (i.e., separating mass interference and inverting the charge polarity from the negative to the positive) obtained for positive secondary ions could be analogically observed with Cs applied for negative ions. The validation of this statement requires further extensive studies, carefully chosen model samples, and gas precursors. It should be highlighted that the improper choice of supplementary gas for a given material and ion detection mode can lead to deteriorated signals compared to results obtained without any gas. For example, it is not advised to use fluorine gas in the negative ion detection mode since F has been shown to decrease the amount of produced negative secondary ions [22].

Moreover, the use of non-standard gas precursors should be discussed with a FIB/SEM manufacturer in advance, regarding potential chamber contamination, corrosive processes, and safety issues (such as health hazards). Unapproved use of new gas precursors can also result in losing an instrument’s warranty.

Another aspect, which is expected to be further investigated, is the use of various primary ion beams. So far, all reported results of combining FIB-TOF-SIMS with GIS were obtained with a continuous monoisotopic ^69^Ga^+^ primary ion beam. However, current FIB/SEM manufacturers offer other primary ion beams, such as Ne, He, Xe, O, and N. Furthermore, dedicated standalone TOF-SIMS instruments commonly operate using a Bi_3_^2+^ primary ion beam. The overview of other primary ion beams used for SIMS can be found in the work of Massonnet and Heeren [191]. This includes ion cluster beams (C_60_^+^, Au_3_^+^, Au_400_^4+^, SF_5_^+^, Bi_3_^+^, water cluster, and argon cluster) as well as metal ion beams (Ga^+^ and In^+^).

It should be noted that both the primary ion beam and supplementary gas modify the matrix effect and, therefore, the ionization efficiency. Therefore, it is not guaranteed that the combination of these two aspects may induce the beneficial chemical state. To verify the optimal experimental conditions, i.e., leading to the maximized ion yield in a given ion detection mode, it is recommended to conduct experiments without any gas (i.e., reference measurements) for various primary ion beams in the two (positive and negative) ion detection modes and then to repeat the experiments with various supplementary gases. For each gas, a fresh set of samples should be ensured to prevent cumulative effects resulting from surface contaminations by a previous gas.

The co-injection of fluorine gas during FIB-TOF-SIMS shows potential for solving the main drawbacks of the technique, such as mass interference and the impossibility of measuring sample components preferentially ionizing with opposite charges. However, no studies involving fluorine have been conducted so far to verify its potential for solving quantification issues. There are several reports [25,26,192,193,194,195] demonstrating the use of MCs^+^ clusters for enabling quantification during SIMS. Using the aforementioned analogy between F and Cs regarding the extreme values of electronegativity/electropositivity, one can hypothetically assume the probability of quantifying chemical data with EF^−^ clusters (where E is an element composing a sample).

A further subject requiring special attention regards the field of semiconductors (in particular Si, Ge, and As). These elements produce both positive and negative ions during Ga^+^ primary ion beam bombardment. Investigating the behavior of semiconductors in the presence of various supplementary gases (ideally F and Cs) could potentially deliver important insights for broadening the understanding on ionization mechanisms. For example, van Heide reported that both positive and negative secondary ion yields are increased in the presence of oxygen; however, the introduction of cesium leads only to the enhancement of Si- secondary ion generation and causes suppression of Si^+^ secondary ions [2]. The latter is consistent with our hypothesis of analogical (to fluorine) potential of Cs to induce charge inversion of generated secondary ions from positive to negative.

Last but not least, the functionality and practical aspects of combining FIB-TOF-SIMS with GIS must be verified. As discussed, the appropriate choice of analysis, primary ion beam, as well as gas precursor, must be ensured. Moreover, the amount of the delivered gases can significantly modify the population of generated secondary ions, i.e., more delivered gas does not necessary translate to higher ionization yields. For example, previous studies [23,151,152] have shown that in the case of Au, the minimum work function of the surface *Φ_0_* is achieved with a Cs coverage of *Θ_min_* ≈ 0.6 atomic layers. Supplying more Cs atoms to the sample’s surface results in a decreased Au ion yield. Additionally, during gas-assisted FIB-TOF-SIMS, the scan area must be sufficiently large, with respect to the expected depth of a sputtered crater. Too deep craters with too small lateral sizes can lead to inhomogeneous delivery/distribution of the supplementary gas at the bottom of the crater, potentially leading to non-uniform material sputtering and, in turn, experimental artifacts. These can be recognized by observing bent structures (for example, bent lines instead of flat ion distributions in the case of planar thin films) close to crater edges in the generated 2D chemical maps in the *x-y* plane.

In summary, there is still plenty of room for exploring the potential, application scope, and optimization of gas-assisted FIB-TOF-SIMS.

## 4. Conclusions

This review summarizes recent advances in gas-assisted FIB-TOF-SIMS conducted with HV-compatible TOF detectors integrated within a FIB/SEM and combined with homemade or commercial gas injection systems. The tremendous potential of this methodology was proven thanks to extensive dedicated studies performed on customized and specialized model samples. Significant enhancements of secondary ion generation, improvement of both the lateral and depth resolution, higher-quality chemical images, separation of mass interference, and capability to induce charge inversion from negative to positive are the main highlights of the developed methodology. Consequently, these new properties open up new opportunities for material chemical characterization, which are inaccessible under standard vacuum conditions without supplementary gases (or demand expensive dedicated instrumentation operating under UHV). Furthermore, the gas-induced efficiency improvement of the ionization process allows for better detection of trace elements and contaminants, which is particularly important in the case of analyzing complex chemical systems, such as Li-ion solid-state batteries.. The ease of using commercial GIS and the possibility of testing a broad spectrum of different gas precursors leave plenty of room for further studies in this field and certainly show great potential not only for upgrading existing experimental systems but also for delivering new insights into the complicated mechanisms of ionization processes and matrix effects.

The principles of TOF-SIMS, data acquisition, representation, and interpretation, as well as technique artifact recognitions, which are discussed in this review, are essential in guiding readers through using this method, as well as to help TOF-SIMS users to avoid typical mistakes when conducting experiments or handling data.

## Figures and Tables

**Table 1:** Advantages and disadvantages of the TOF-SIMS technique.

Advantages	Disadvantages
Representation of a material’s chemical structure in 3D with nanoscale resolution Parallel detection of all types of generated ions (light and heavy) Detection of single ions and ionized molecules Recognition of isotopes High lateral resolution (<50 nm) High depth resolution (<10 nm) High sensitivity (ppm-ppb) No sample preparation required prior to measurement (samples have to be flat and conductive) Qualitative technique Wide range of analysis area (1–10^4^ µm) Ion-induced secondary electron (SE) imaging of the surface’s nano- and microstructure	Ionization efficiency determined by the matrix effect Quantification is not possible (or difficult to assess) Potential mass interference (which can lead to data misinterpretation) Destructive technique Either positive or negative ions can be collected during a single experiment from a given volume Topography sensitivity Ionization efficiency of some elements might be too low to represent their distribution in 2D or 3D Initial knowledge of the sample’s composition is needed for data interpretation (due to mass interference) TOF-SIMS signals can be influenced by the sample’s crystallinity [3]

Note that different TOF-SIMS detectors have different lateral resolution, depth resolution, mass resolution, as well as sensitivity. Spatial resolution depends on the primary ion beam type and parameters: The highest lateral resolution is obtained at high primary ion beam energies (usually 30 keV), and the highest depth resolution is obtained at low primary ion beam energies (usually ≤ 5 keV). Furthermore, nonconductive samples can be measured with dedicated TOF-SIMS instruments when applying an electron flood gun for charge compensation.

**Table 3:** Summary of gas enhancement factors for TOF-SIMS signals, *GAE_TOF-SIMS_*, and gas enhancement factors for sputter rates, *GAE_SP_*, obtained in the presence of water vapor and fluorine gas [21,22,66].

Element	*GAE_TOF-SIMS,water_vapor_* [-]	*GAE_TOF-SIMS,fluorine_* [-]	*GAE_SP,water_vapor_* [-]	*GAE_SP,fluorine_* [-]
^7^Li^+^ (in Au/Li_7_La_3_Zr_2_O_12_/Pt/MgO/Si)		(8.9 ± 0.3) ^(b)^ [22] (10.9 ± 0.5) ^(c)^ [22]		
^24^Mg^+^ (in Au/Li_7_La_3_Zr_2_O_12_/Pt/MgO/Si)		(5.4 ± 0.2) ^(b)^ [22] (8.4 ± 0.5) ^(c)^ [22]		
^27^Al^+^ (in Zr Al)	(2.7 ± 0.2) ^(a)^ [66]	(2.6 ± 0.3) ^(a)^ [66]	(0.54 ± 0.03) ^a)^ [66]	(1.9 ± 0.1) ^(a)^ [66]
^28^Si^+^ (in Au/Cr/SiO_2_/Si)		(5± 3) ^(b)^ [66] (11 ±6) ^(c)^ [66]		
^28^Si^+^ (in ZrSi)	(3.4 ± 0.7) ^(a)^ [66]	(3.8 ± 0.8) ^(a)^ [66]	(3.8 ± 0.8) ^(a)^ [66]	(3.8 ± 0.8) ^(a)^ [66]
^28^Si^-^ (in Au/Cr/SiO_2_/Si)		(0.0062 ± 0.0003) ^(b)^ [22] (0.018 ± 0.002) ^(c)^ [22]		
^52^Cr^+^ (in Au/Cr/SiO_2_/Si)		14 ± 2) ^(b)^ [22] (15 ±2) ^(c)^ [22]		
^52^Cr^-^ (in Au/Cr/SiO_2_/Si)		(0.31 ± 0.02) ^(b)^ [22] (1.37 ± 0.09) ^(c)^ [22]		
^63^Cu^+^	(10 ± 2) ^(a)^ [21]	(510 ± 80) ^(a)^ [21]	(0.9 ± 0.2) ^(a)^ [21]	(0.6 ± 0.2) ^(a)^ [21]
^63^Cu^+^ (in ZrCu)	(15.1 ± 0.3) ^(a)^ [66]	(176 ± 15) ^(a)^ [66]	(0.69 ± 0.02) ^(a)^ [66]	(1.89 ± 0.08) ^(a)^ [66]
^90^Zr^+^	(0.18 ± 0.08) ^(a)^ [21]	(0.73 ± 0.03) ^(a)^ [21]	(6 ± 2) ^(a)^ [21]	(2.3 ± 0.2) ^(a)^ [21]
^90^Zr^+^ (in ZrAl)	(2.21 ± 0.06) ^(a)^ [66]	(1.18 ± 0.06) ^(a)^ [66]	(0.54 ± 0.03) ^(a)^ [66]	(1.9 ± 0.1) ^(a)^ [66]
^90^Zr^+^ (in ZrSi)	(1.29 ± 0.07) ^(a)^ [66]	(5.34 ± 0.05) ^(a)^ [66]	(0.75 ± 0.04) ^(a)^ [66]	(1.77 ± 0.08) ^(a)^ [66]
^90^Zr^+^ (in ZrCu)	(3.1 ± 0.2) ^(a)^ [66]	(0.81 ± 0.05) ^(a)^ [66]	(0.69 ± 0.02) ^(a)^ [66]	(1.89 ± 0.08) ^(a)^ [66]
^90^Zr^+^ (in Au/Li_7_La_3_Zr_2_O_12_/Pt/MgO/Si)		(12.4 ± 0.3) ^(b)^ [22] (20.7 ± 0.5) ^(c)^ [22]		
^107^Ag^+^	(6.7 ± 0.8) ^(a)^ [21]	(350 ± 20) ^(a)^ [21]	(1.2 ± 0.2) ^(a)^ [21]	(2.1 ± 0.3) ^(a)^ [21]
^184^W^+^	(2.1 ± 0.3) ^(a)^ [21]	(0.170 ± 0.008) ^(a)^ [21]	(1.0 ± 0.1) ^(a)^ [21]	(2.3 ± 0.3) ^(a)^ [21]
^139^La^+^ (in Au/Li_7_La_3_Zr_2_O_12_/Pt/MgO/Si)		(2.42 ± 0.06) ^(b)^ [22] (2.71 ± 0.07) ^(c)^ [22]		
^195^Pt^+^ (in Au/Li_7_La_3_Zr_2_O_12_/Pt/MgO/Si)		(3400 ± 80) ^(b)^ [22] (2150 ± 50) ^(c)^ [22]		
^197^Au^+^ (in Au/Cr/SiO_2_/Si)		(1990 ± 70) ^(b)^ [22] (630 ± 20) ^(c)^ [22]		
^197^Au^-^ (in Au/Cr/SiO_2_/Si)		(0.116 ± 0.005) ^(b)^ [22] (0.39 ± 0.01) ^(c)^ [22]		
^197^Au^+^ (in Au/Li_7_La_3_Zr_2_O_12_/Pt/MgO/Si)		(730 ± 20) ^(b)^ [22] (257 ± 7) ^(c)^ [22]		

Note the different criteria of calculating *GAE_TOF-SIMS_*: ^(a)^ Based on depth profiles, ^(b)^ based on signal integrals in the mass spectra, and ^(c)^ based on signal maxima in the mass spectra.

**Table 4:** Advantages and disadvantages of fluorine gas-assisted FIB-TOF-SIMS.

Advantages	Disadvantages
Enhancement of positive secondary ion generation by up to two to four orders of magnitude Significant increase in lateral and depth resolution in a single measurement Improved quality of depth profiles, 2D and 3D chemical images Separation of mass interference Inversion of generated secondary ion polarity from negative to positive Enabled direct and complete information on positively and negatively ionizing metals from the same analytical volume Increased sputter rates	Not suitable for studying negative ions Potential new mass interference scenarios with F Modified efficiency of metal oxide and metal hydride formation Non-uniform sputtering of a crater button in the case when the crater depth is too large compared to the lateral size Not suitable for samples containing F (not possible to distinguish secondary F ions originating from the sample and the gas precursor) Injection of XeF_2_ to the analytical chamber contaminates the surface of other samples mounted on the stage In current commercial GIS, it is not possible to control the amount of delivered gas, and the dose of injected gas cannot be directly measured (comparable studies should be performed at approximately the same time ensuring comparable experimental conditions)
